# To Correspondents

**Published:** 1844-06-01

**Authors:** 


### THE MEDICAL EXAMINER.

PHILADELPHIA, JUNE 1, 1844.

### TO CORRESPONDENTS.

In order to render the pages of the Examiner as interesting as possible, the Editor deems it necessary that each number shall embrace a variety of topics; consequently, he is compelled to avoid the insertion of long essays on any one subject, and especially such as abound in details and references which are not absolutely necessary for a proper understanding of the subjects on which they are written. While, therefore, he solicits from his correspondents and subscribers early accounts of interesting cases, epidemic and endemic diseases, and remarks on subjects of interest to practitioners generally, he requests that their communications may be prepared with as much brevity as is consistent with a clear exposition of the objects in view. Valuable papers are sometimes necessarily laid aside because of their great prolixity.

---

The following communication relates to matters of interest, not only to the Hospitals and Medical Teachers in Philadelphia, but to Students of Medicine and the profession at large. We had hoped that our friend of the Bulletin of Medical Science would have seen the necessity, after the use that has been made of his remarks, of correcting his own mistake, and so putting others right whom he has inadvertently misled. He has undoubtedly fallen into a great error in supposing that the clinics at the colleges—except on the occasion of some rare *displays*—have lessened the attendance of students at the hospitals. On the contrary, we have reason for believing that at no time in the history of Philadelphia were these institutions more resorted to, or the zeal and ability of the medical officers more energetically displayed, than within the last two or three years. The clinics at the colleges are not substituted for those at the hospitals, as some seem to suppose, but are in addition thereto. The consequence is that a multitude of cases are now exhibited and prescribed for, in the presence of the classes that attend at the Philadelphia schools, that never would appear in the wards of a hospital.

To the Editor of the Medical Examiner.

SIR,—I see it stated in certain of the medical journals of this country, on the authority of the *Bulletin of Medical Science*, published in this city, that the opportunities for clinical instruction at the Philadelphia Hospital were less numerous during the last session than previously. Can this be true? and is not the contrary the fact? A few years ago, but one course of lectures was delivered there—now there are two; and although most of the students of the University of Pennsylvania attend their own professors only; and those of the Jefferson Medical College theirs; it must be borne in mind, that the ticket admits them to both, although it includes omnibus fare for one only. The opportunities for clinical instruction are, in reality, twice as numerous there as they were five years ago; and whatever objection may be made to the new system of clinical teaching, in surgery especially, connected more immediately with the two medical schools, it cannot be contested, that opportunities for such instruction are more frequent and various now than they have ever been. Where classes are numerously attended, the difficulties in the way of a proper plan for clinical instruction are weighty: they are forcibly felt by the parties concerned; yet because such difficulties exist every where, it is not just or commendable to depict them in such a manner as to make it appear that they apply to Philadelphia exclusively; and to underrate,—doubtless, from ignorance of the true state of the case, with which the editor might, however, have readily made himself acquainted,—the eminent advantages that Philadelphia possesses as a seat of clinical instruction. Let any evil that exists be corrected, but certainly not exaggerated.

ARMOSTER.

*Philadelphia, May* 24, 1844.

---

We have received the second number of the *Illinois Medical and Surgical Journal*, a new monthly publication, from the press of Ellis and Fergus, Chicago, Ill. It is under the editorial management of Dr. Blaney, one of the Professors of the Rush Medical College, recently established in that place. The commencement of such a work in that remote section, betokens a spirit of enterprise in our western brethren worthy of success.

